# Enhancement of Antimicrobial and Dyeing Properties of Cellulosic Fabrics via Chitosan Nanoparticles

**DOI:** 10.3390/polym14194211

**Published:** 2022-10-07

**Authors:** Rehab M. Mosaad, Mona H. Alhalafi, El-Amir M. Emam, Marwan A. Ibrahim, Hassan Ibrahim

**Affiliations:** 1 Department of Biology, College of Science, Majmaah University, AL-Majmaah 11952, Saudi Arabia; 2 Faculty of Women for Arts, Science and Education, Ain Shams University, Cairo 11566, Egypt; 3 Department of Chemistry, College of Science, Majmaah University, AL-Majmaah 11952, Saudi Arabia; 4 Faculty of Applied Arts, Textile Printing, Dyeing and Finishing Department, Helwan University, Cairo 11795, Egypt; 5 Pretreatment and Finishing of Cellulosic Fibers Department, Textile Research and Technology Institute, National Research Centre, 33 El-Behouth St., Dokki, Cairo 12622, Egypt

**Keywords:** cellulosic fabrics, chitosan nanoparticles, acid dyes, antimicrobial activity of dyeing affinity, cytotoxicity assay

## Abstract

The primary goal of this study is to prepare chitosan nanoparticles (CSNPs) by the ionic gelation method via the treatment of chitosan (0.2 wt.%) with tripolyphosphate (0.2 wt.%) ultrasonically for 45 min. FT-IR spectroscopy and TEM images were used to characterize and validate CSNP production. Cellulosic materials with different concentrations of CSNPs have better antibacterial and colouring characteristics. The treated cellulosic fabrics were analyzed by FT-IR spectroscopy, SEM, and thermogravimetric analysis. Colourimetric data measurements expressed in K/S values were used to evaluate the impact of CSNPs on the dyeing affinity of cellulosic materials. In addition, antibacterial activity against bacteria and fungi was tested on the treated cellulosic fabrics. According to the K/S values, cellulosic textiles treated with CSNPs (0.3 wt.%) had a better affinity for acid dyeing. These textiles also offer better antibacterial properties and are more resistant to washing, light, and rubbing. A cytotoxicity study found that CSNPs give cellulosic materials antibacterial and acid dyeing properties, which is good for the environment.

## 1. Introduction

Cotton fabrics are commonly utilized in the textile industry because they contain cellulose (96%). Cotton fabrics also have excellent chemical and physical qualities, such as stability, water absorption, and comfort [1,2].

Cellulosic fabrics have a moderate affinity for reactive and direct dyes in the presence of salts to increase their dye exhaustion, but they don’t have any affinity for acid dyes [3,4,5]. The addition of salts restricts the penetration of dyes inside cellulosic fabrics, causing pollution of running water [6,7,8]. On the other hand, acid dyes can replace direct and reactive dyes for cellulosic dyeing because it is cheaper and less polluting than running water. This replacement can be made via chemical modification of these fabrics, such as the amination process [9,10]. The amination process is accompanied by the depolymerization of cellulosic chains. Therefore, there is great attention to replacing these chemical modification processes in finished cellulosic fabrics with cationized agents to improve their dyeability and antibacterial activity [11,12].

Cationization is the chemical modification of cotton that results in the formation of cationic (positively charged) dyeing sites in place of existing hydroxyl (-OH) dyeing sites [13]. Lei and Lewis reviewed a wide range of substances that can be used to prepare cationic sites in cotton fabric, including glycidyl trimethylammonium chloride, N, N-dimethylazetidinium chloride, N-methylol acrylamide, chloro propionyl chloride, polyepichlorohydrin acrylamide, and nicotinyl thioglycolate [13,14]. The most common cactionizing agents used for cellulose-based materials are quaternary ammonium compounds but they have a range of health effects such as coma, convulsions, hypotension, and death. In addition, these cationic agents are toxic and cause huge environmental problems. Biopolymers such as chitin, chitosan, and gelatin can be used to overcome these risks [3,15].

Chitosan (CS) and chitosan nanoparticles (CSNPs) are two biopolymers that can be used to make completed cellulosic textiles. Because of its biocompatibility, biodegradability, and non-toxicity, CS offers a wide range of biological uses, including medication administration and wound healing [16,17,18,19,20]. CSNPs have a high surface area and zeta potential stability. Therefore, CSNPs have higher biological activity than CS [21,22,23,24,25], especially in drug, gene, and vaccine delivery [26,27,28,29,30,31,32,33,34,35,36,37]. Therefore, CSNPs can be used to enhance cellulosic dyeability and antimicrobial activity more than CS itself [38,39,40].

In the present work, chitosan nanoparticles were synthesized by ionic gelation of chitosan and tri-polyphosphate. Then, CSNPs were used to improve both the dyeability and antimicrobial activity of cellulosic fabrics. Cellulosic fabrics treated with CSNPs were dyed with two acid dyes. TEM, FT-IR spectra, SEM, and TGA were used to characterize the synthesized CSNPs and their treated cellulosic fabrics. Colourimetric data was used to evaluate the dyeability of fabrics and their fastness properties. The antimicrobial activity and cytotoxicity assay together were evaluated to illustrate the green effect of CSNPs as antimicrobial without toxicity.

## 2. Materials and Methods

### 2.1. Materials

Chitosan (CS) (Aldrich, viscosity 1860cps, molecular weight; 100,000–300,000, degree of deacetylation 79.0%). Sodium hydroxide (Modern Lab chemicals, Cairo, Egypt), monochloroacetic acid (Fluka, CAS No: 79-11-8, EINECS No: 201-178-4), Penta sodium tri poly phosphate (TPP); Fluka were used without further purification. Scoured and bleached 100% cotton was used. Hostapal^®^ CVL-ET (nonionic wetting agent based on alkyl aryl polyglycol ether, Clariant).

Dyes: Acid Blue 317^®^ (AB) and Acid Red 88^®^ (AR), Single azo, (OHYOUNG Industrial Co., Ltd.) were used.

### 2.2. Methods

#### 2.2.1. Preparation of Chitosan Nanoparticles (CSNPs)

Chitosan nanoparticles were prepared based on the modified ionotropic gelation method [30]. Briefly, Chitosan was dissolved in 1% (v/v) acetic acid and left stirring for 24 h, and the pH was adjusted to pH 5.5 with 0.01 N NaOH. TPP was dissolved separately in deionized water until the final concentration was 0.1 mg/mL. Then, drop by drop, the TPP solution was added to the chitosan solution at different ratios of TPP to chitosan. This was done at room temperature with a strong magnetic stirrer. The resulting suspension was then left under ultrasonication for 45 min.

#### 2.2.2. Finishing of Cellulosic Fabrics with Chitosan Nanoparticles

**Figure 1:**
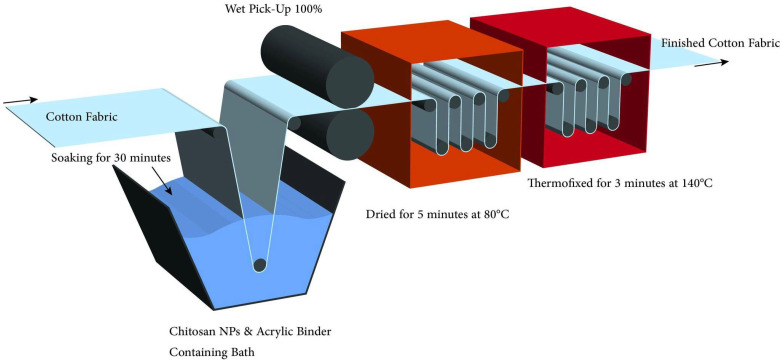
Finishing of cellulosic fabrics with chitosan nanoparticles via pad-dry-cure process.

The pad-dry-cure process was used to treat cotton fabrics with chitosan nanoparticles. For all treatments, 30 cm × 30 cm of fabrics were soaked for 30 min in a CSNPs solution containing acrylate binder (1%) and then passed through a padded mangle with a 100-percent wet pick-up. The materials were then dried for five minutes at 80 °C before being thermofixed for three minutes at 140 °C. Finally, cotton materials were washed and dried so that they could be characterized, tested for bacteria, and treated further. This process can be shown in Figure 1.

#### 2.2.3. Procedures for Using Two Acid Dyes in Fabric Dyeing

**Figure 2:**
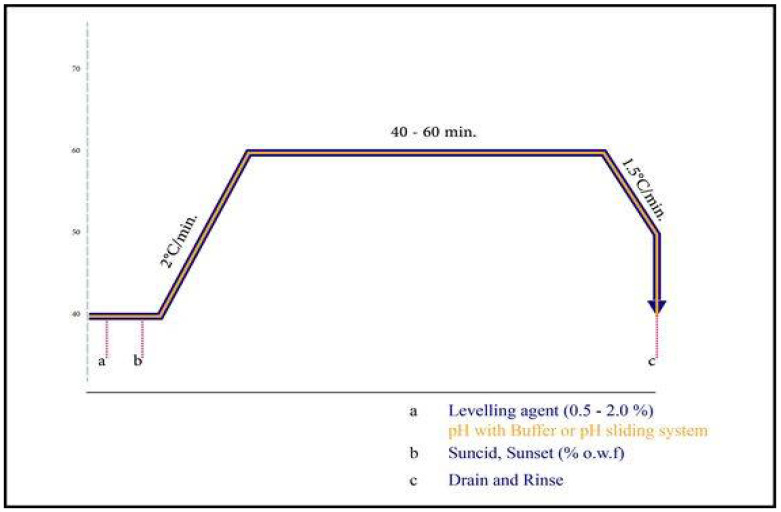
Processing dyeing curve of the two commercial acid dyes used: A: Levelling agent, 0.5–2.0%, PH with Buffer or PH sliding system; B: Dye; C: Drain and Rinse.

A common procedure was used to dye the treated and untreated cotton fabrics with acid dyes of a five-weight solution% dye (o.w.f.) and 5% wt. In this experiment, 100-percent acetic acid was used. The dyeing process began at 40 °C and gradually increased to 100 °C for 30 min, with dyeing taking place at 100 °C for 40–60 min at a material-to-liquor ratio of 1:50. The materials were completely washed with 1–5 g/L non-ionic detergent for 30 min at 60 °C after dyeing, and then rinsed with cold water. Fabrics that had been dyed were dried (Figure 2).

### 2.3. Characterization

#### 2.3.1. FT-IR Spectra

The FT-IR spectra of the samples were recorded by using an FT-IR spectrophotometer (Nexus 670, Nicolet, USA) in the region of 4000–400 cm^−1^ with a spectra resolution of 4 cm^−1^.

#### 2.3.2. Transmission Electron Microscopy (TEM)

The shape and size of chitosan nanoparticles (CSNPs) were practically obtained by using TEM, JEOL-JEM-1200, USA. Specimens for TEM measurements were prepared by placing a drop of colloidal solution on 400 mesh copper grids coated with an amorphous carbon film and evaporating the solvent in the air at room temperature. The average size of the prepared CSNPs was found by looking at the size of 100 nanoparticles in enlarged microphotographs. These 100 nanoparticles were found in several randomly chosen areas.

#### 2.3.3. Thermo Gravimetric Analysis (TGA)

Thermo gravimetric analysis (TGA) was performed at a temperature starting from 25 °C to 600 °C under an inert nitrogen atmosphere with a heating rate of 10 °C min^−1^ using the instrument: SDT Q600 V20.9 Build 20, USA.

#### 2.3.4. Mechanical Properties

The mechanical properties expressed in tensile strength (TS) of the fabric sample were determined by the ASTM Test Method D5035, with a Q-Test 1/5 tensile tester. Three specimens for each treated fabric were tested in the warp direction, and the average value was recorded to represent the fabric breaking load (Lb).

#### 2.3.5. Colour Measurements

On the Ultra Scan Pro in the Hunter lab, the whiteness index (WI) and yellowness index (YI) of treated and untreated cotton fabrics were measured. The ISO 105-CO2:1989 test technique was used to determine colour fastness to washing. The washing fastness tests were carried out in an ATLAS launder metre (Germany) at 50 °C for 45 min using a 5 g/L nonionic detergent. Using a carbon arc lamp, the light-fastness was tested according to ISO 105-B02: 1988. The test method ISO 105-X12: 1987 40 was used to determine rubbing fastness. A difference-in-colour formula E CIE (L*, a*, b*): E CIE (L*, a*, b*): E CIE (L*, a*, b*) was used. The Hunter-Lab spectrophotometer was used to quantify the total difference E CIE (L*, a*, b*) (model: Hunter Lab DP-9000). The following formula can be used to determine the colour strength:

$$Relative\;colour\;strength=\frac{K/S\;of\;treated\;samples}{K/S\;of\;untreated\;samples}\times 100\;$$

### 2.4. Antimicrobial Activity Evaluation

#### 2.4.1. Materials

Gram-positive bacteria included *S. aureus*, ATCC 6538, and *B. subtilis*, ATCC 6633, whereas gram-negative bacteria included *E. coli*, ATCC 11229, and *Proteus*, ATCC 33420. The fungal strains employed were *Aspergillus Niger*, ATCC 13497, and *Candida*, ATCC 10231. These bacterial and fungal strains were selected as test cells because they are the most frequent bacteria in the wound infection. Fresh inoculants for antibacterial assessment were prepared in nutrient broth at 37 °C for 24 h. Antibiotics were used as reference drugs, such as Ampicillin used as a reference for gram-positive bacteria and ciprofloxacin used for gram-negative bacteria and fungi.

#### 2.4.2. Test Methods

All antimicrobial activity tests were done in triplicate to ensure reproducibility. The disc diffusion method [41] was used for assessing the antibacterial activity of dyed fabrics antimicrobial activity. Briefly, discs of 10 mm diameter were cut from dyed fabrics. Nutrient agar plates were inoculated with microbial culture. The cut discs of dyed fabrics were placed onto the surface of inoculated plates. The plates were incubated at 37 °C for 48 h. The inhabitation zone (distance from disc circumference in mm) was determined for each disc. In the antibacterial assay, all data were the means from at least three parallel experiments and the discrepancies among them were less than 5%.

### 2.5. Chitosan Nanoparticles Cytotoxicity Test Assay

The cells were subcultured with 0.15 percent trypsin versene and maintained in DMEM with 10% foetal bovine serum at 37 °C, 5% CO_2_, and 95% humidity. Skin cell line (BJ-1) “Immortalized normal foreskin fibroblast cell line” (Stockholm, Sweden) was used. Cells extra: Vacsera made them (Giza, Egypt).

After 24 h, the medium was changed to a serum-free medium with 100 g/mL extracts in triplicates. We gave the cells doxorubicin (100 g/mL) and distilled water for 24 h. Using the MTT (3,4,5-dimethylthiazol-2-yl)-2,5-diphenyltetrazolium bromide) test [42].

Percentage cytotoxicity=(1−(Av(x)/(Av(NC)))×100

where Av: average, x: absorbance of the sample well measured at 595 nm with reference 690 nm, and NC: absorbance of negative control measured at 595 nm with reference 690.

## 3. Results

### 3.1. Synthesis of Chitosan Nanoparticles

Ionic gelation was used to synthesize chitosan nanoparticles from chitosan and tri poly phosphate, followed by 45 min of ultrasonication to convert the -NH_2_ groups in chitosan to -NH_3_^+^ [43,44,45,46,47,48]

**Figure 3:**
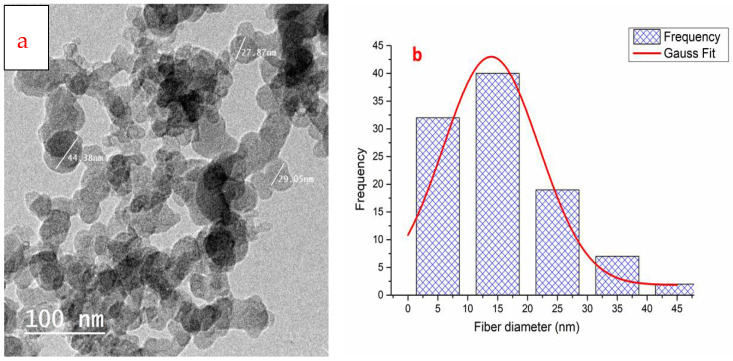
TEM image (**a**); histogram of the particle size distribution of synthesized chitosan nanoparticles (CSNPs) using a chitosan concentration of 0.2 weight percent and 0.1 weight percent TPP for 45 min ultrasonication and a pH value of 5.5 (**b**).

Figure 3a shows a transmission electron microscope image of the manufactured chitosan nanoparticles using a chitosan concentration of 0.2 wt. percent and 0.1 wt. percent TPP for 45 min at a pH value of 5.5. Figure 3b shows a histogram of the particle size diameter of CSNPs and from the histogram, most CSNPs particles size ranged from 5–35nm.

**Figure 4:**
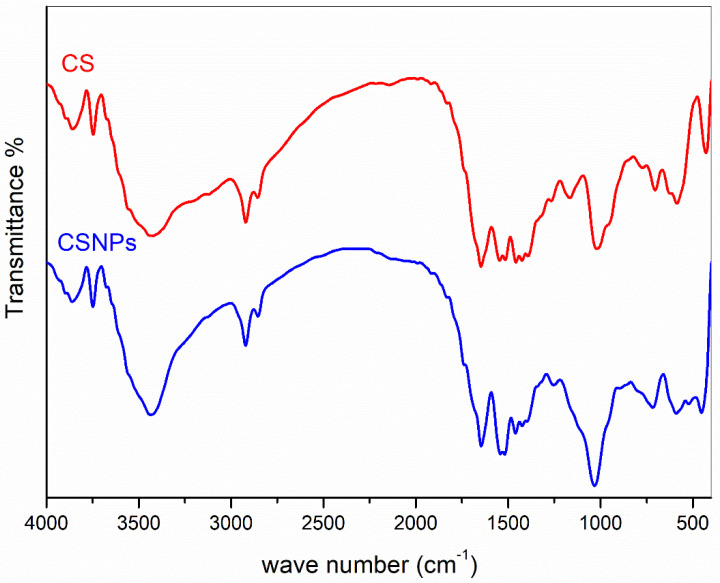
FT-IR spectra of chitosan and prepared chitosan nanoparticles.

Figure 4 shows the FT-IR spectra of chitosan (CS) and chitosan nanoparticles (CSNPs). Both CS and CSNPs show the same band peaks at 3434 cm^−1^ for amino groups and two at 1637 cm^−1^ and 1564 cm^−1^ for amide I and amide II [22,49]. However, CSNPs show wider band peaks with bathochromic shifts [37,50].

### 3.2. Chitosan Nanoparticles as a Finishing Agent for Cellulosic Cotton Fabrics and their Dyeing with Two Acid Dyes

The influence of chitosan nanoparticles (CSNPs) on the mechanical properties of cellulosic fabrics is shown in Table 1. The mechanical properties of treated fabrics and treated and coloured fabrics are slightly enhanced. This surprising gain in mechanical characteristics is a result of the nanostructure of CSNPs, their penetration into cellulosic textiles, and the increased crosslinking of cellulosic fabrics and CSNPs [51]. Due to the nanostructure of chitosan nanoparticles, treatment of cellulosic cotton fabrics results in an increase in the whiteness index (WI) and a decrease in the yellowness index (YI) (CSNPs).

**Figure 5:**
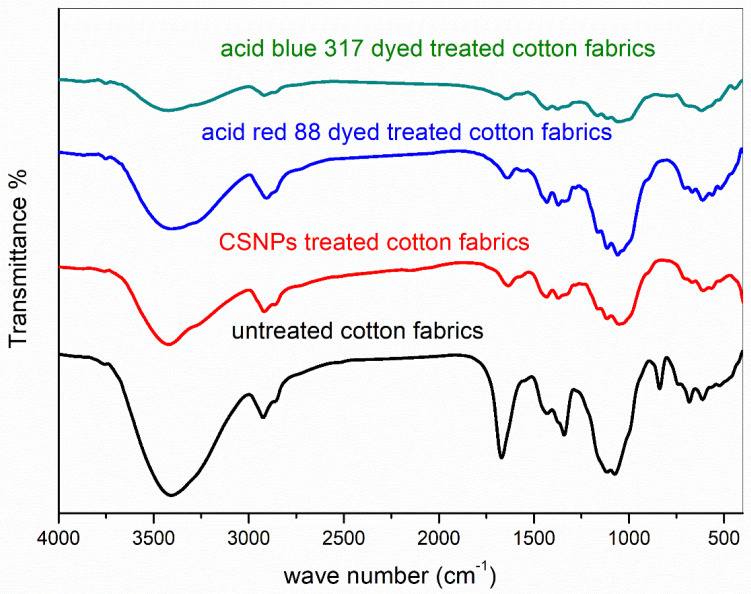
FT-IR spectra of untreated cotton fabrics, cotton fabrics treated with CSNPs, Acid Blue 317 dyed and treated cotton fabrics, and cotton fabrics dyed and treated with Acid Red 88.

Figure 5 shows the FT-IR spectra of untreated cotton fabric, cotton fabric treated with chitosan nanoparticles (CSNPs), and cotton fabric treated and coloured. The FT-IR spectrum of untreated cotton fabrics reveals typical band peaks for the OH and CH stretching groups at 3340 and 2900 cm^−1^, respectively, as well as bands at 1648, 1428, and 1057 cm^−1^ for the OH and CH stretching groups. [52]. Cotton fabrics treated with CSNPs exhibit a broader absorption band peak at 3434 cm^−1^ for NH stretching in amine and amide, which is caused by hydrogen bonds between amino and hydroxyl groups in both nano chitosan and cellulose molecules; there are two peaks at 1637 cm^−1^ and 1564 cm^−1^ for amide I (C=O) and amide II (N–H), respectively, and a peak at 1383 cm^−1^ for C–CH_3_ vibration. [49,50]. As a result, the FT-IR analysis confirms that cotton fabrics have been chemically modified with chitosan nanoparticles. The combined bands of cotton, CSNPs, and two dyes are visible in the FT-IR spectra of treated and coloured cotton fibres. Additionally, the peaks of two acid dyes exhibit a red shift.

**Figure 6:**
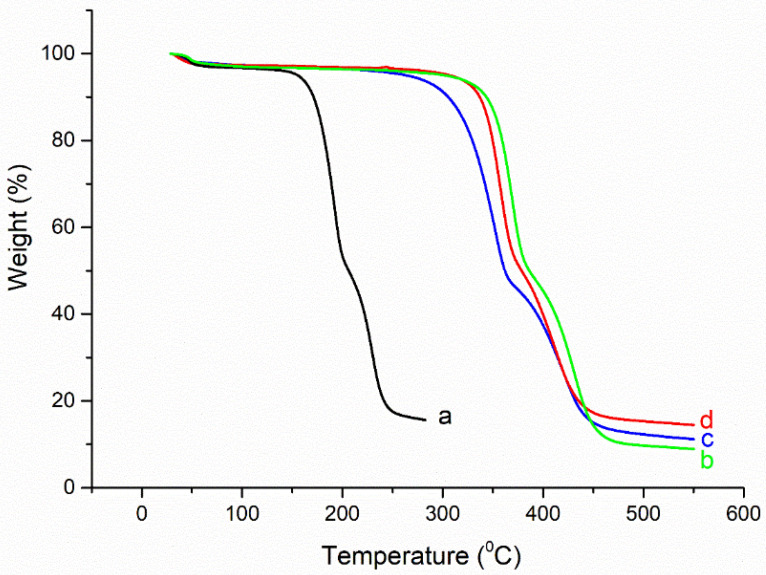
TGA thermograph of untreated cotton fabrics (**a**); CSNPs treated cotton fabrics (**b**); Acid Blue 317 dyed CSNPs treated cotton fabrics (**c**); Acid Red 88 dyed CSNPs treated cotton fabrics (**d**).

Thermal analysis, as described in TGA, has been used to explain the change in weight loss when the temperature is increased gradually from room temperature to the point of total disintegration. Figure 6 compares the thermal behaviour of untreated, treated, and coloured cotton materials under identical conditions. Figure 6 illustrates the three major stages of decomposition in all samples, albeit in a different order. For untreated cotton fabrics, it begins decomposing at temperatures below 320 °C (volatilization of a few M. wt. compounds); continues decomposing at temperatures between 320 and 380 °C (L-glucose synthesis); finally, it carbonizes at temperatures above 400 °C. The treated cotton fabric exhibits the same stages as the untreated cotton fabric but with increased thermal stability due to the presence of chitosan nanoparticles containing amino groups. The same increase in thermal stability is observed for dyed materials that dye both amino groups of CSNPs and N=N for the two acid dyes (see Figure 6).

**Figure 7:**
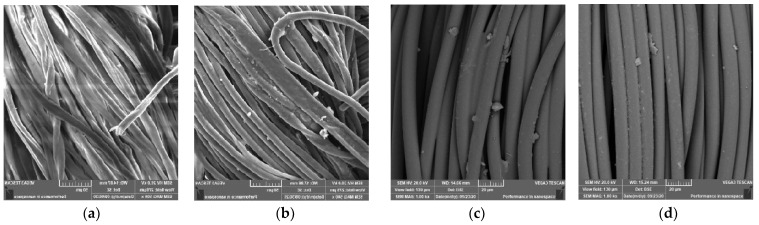
SEM images of: (**a**) untreated cotton fabric, (**b**) treated cotton fabric with CSNPs, (**c**) Acid Red 88 dyed cotton fabric with CSNPs, and (**d**) Acid Blue 317 dyed cotton fabric with CSNPs.

SEM microscopy was used to analyze the surface morphology of treated and coloured cotton fabrics (SEM). Figure 6 illustrates the alteration in the morphology of cotton fibres following treatment with chitosan nanoparticles (CSNPs) and dyeing with two acid colours. Cotton fabrics that have not been treated exhibit a smooth surface (Figure 7a). The smooth surface is embedded with amazing nanoparticles derived from CSNPs on both the inside and outside of the fibre surface (Figure 7b). Cotton fabrics dyed with two acid dyes exhibit coated and granulated CSNPs (Figure 7c,d). Thus, SEM imaging confirms the presence of CSNPs both within and outside cotton fabrics, both completed and finish dyed, as well as the coating process for coloured cotton samples.

**Figure 8:**
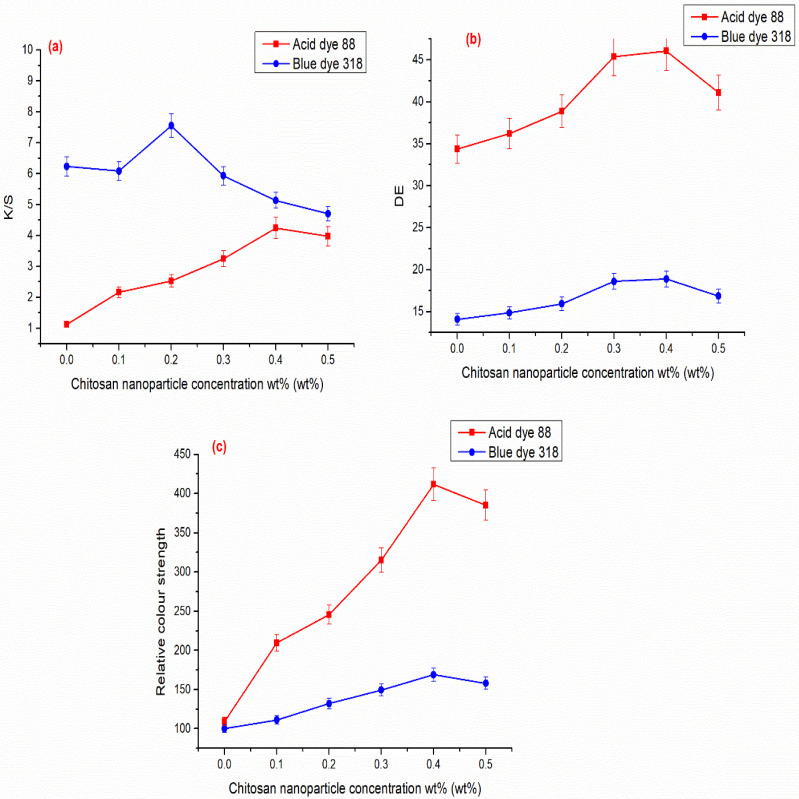
Effect of chitosan nanoparticles on the colour strength of Acid Red 88 and Acid Blue 318 dyes as measured by: (**a**) K/S values; (**b**) E values; (**c**) relative colour strength.

The colour intensity, represented in K/S values, is proportional to the amount of dye absorbed at the cellulosic cotton surface. Acid Blue 318 dye has higher K/S values than Acid Red 88 dye, as seen in Figure 8, because it generates more cationic sites on the surface of cellulosic cotton thanks to CSNPs. Additionally, Figure 8b,c illustrates the similar trend for this increment in colour difference (E) and relative colour intensity between dye-coated and untreated CSNPs. Additionally, Acid Red 88 has a greater ability than Acid Blue 317 to attach to CSNPs and cellulosic cotton without the use of a crosslinker. This is due to higher values for colour difference and relative colour strength. Additionally, Figure 8 shows that the K/S values for two acid dyes increased as chitosan concertation increased.

Additionally, we found an increase in K/S values as the chitosan concentration increased up to 0.2 wt. percent and then a reduction. This is because after reaching that concentration, cellulosic cotton fibres became saturated with chitosan and no longer required any for dyeing. While it is competing with textiles for dye absorption, it is responsible for the reduction in K/S values following that concentration. Additionally, the energy difference exhibits the same trend as K/S for chitosan concentration, which has increased significantly up to 0.2 weight percent chitosan concentration, at which point it will decrease for the same reason.

Table 2 shows that all dyed fabrics possess excellent to very good rubbing, washing, and perspiration fastness resistance after being subjected to 0.2 wt.% nonionic detergent at 88 °C for 30 min. In addition, these fabrics show excellent resistance to light.

### 3.3. The Antimicrobial Activity Study

**Figure 9:**
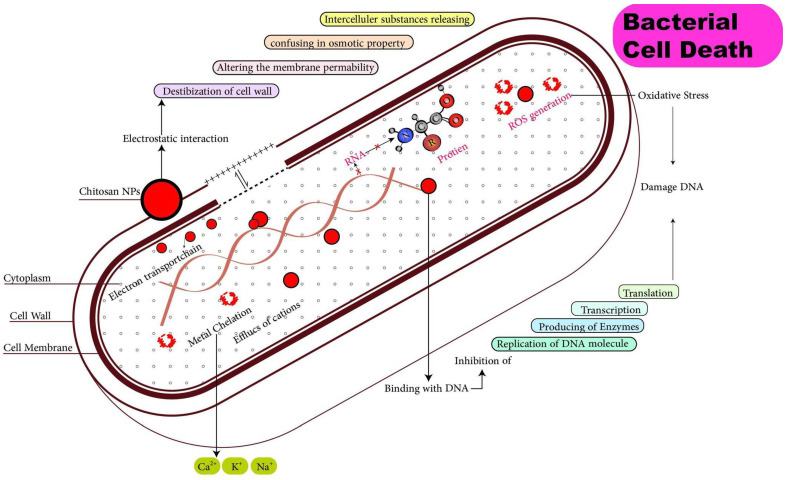
Antibacterial mechanism of chitosan nanoparticles.

In acidic media, chitosan (CS) exhibits cationic properties due to the conversion of NH_2_ to NH_3_^+^. Chitosan’s cationic nature allows it to permeate bacterial cell walls, effectively destroying them [53,54,55]. In addition, CS has the same action on fungi. The primary disadvantage of CS is that it is more active against gram-positive bacteria than against gram-negative bacteria. Thus, chitosan nanoparticles (CSNPs) may be used to address this issue since their nanostructure facilitates their entry into microbial (bacterial and fungal) cells, as shown in Figure 9.

**Figure 10:**
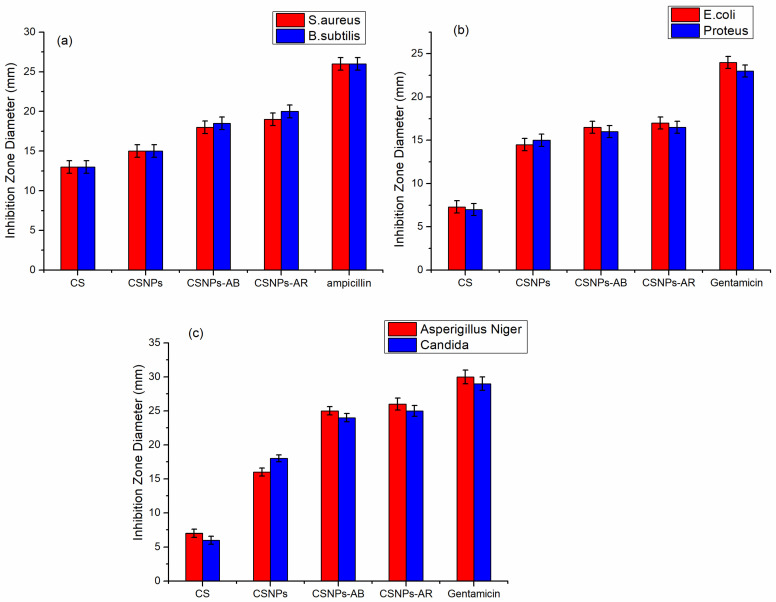
Antimicrobial activity of cotton fabrics treated with chitosan (CS), chitosan nanoparticles (CSNPs), and chitosan nanoparticles dyed Acid Blue 317 and Acid Red 88 against: gram-positive bacteria (**a**), gram-negative bacteria (**b**), and fungi (**c**).

The disc inhibition method was used to assess the antimicrobial activity of cotton fabrics treated with chitosan (CS) and chitosan nanoparticles (CSNPs), treated with CSNPs and dyed with Acid Blue 317 (CSNPs-AB), and treated with CSNPs and dyed with Acid Red 88 (CSNPs-AR), against two gram-positive, two gram-negative, and two fungi. Figure 10 demonstrates that while CS has a greater impact only on gram-positive bacteria, CSNPs have a greater impact on both gram-positive and gram-negative bacteria. Additionally, Figure 10 demonstrates how the inclusion of azo and sulphonic groups in two acid dyes enhances their antibacterial action. The reactive groups of the two acid dyes enhance the antibacterial activity, and the nanostructure of CSNPs overcomes bacterial and fungal structures.

### 3.4. Cytotoxicity of Chitosan Nanoparticles (CSNPs) via MMT Assay

**Figure 11:**
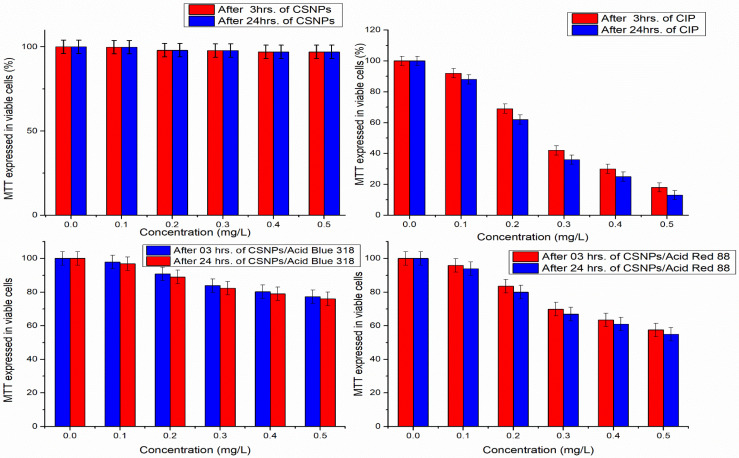
Mitochondrial metabolic activity (MTT) assay expressed in viable cells of chitosan nanoparticles (CSNPs), ciprofloxacin (CIP), CSNPs composite with Acid Red 88 and Acid Blue 318 after 3 and 24 h, cell culture.

The main goal of this study was to use safe finishing agents to enhance cotton fabrics’ dyeing ability with acid dyes. Chitosan has been confirmed that it is non-toxic and biocompatible with tissues [56,57]; the cytotoxicity of chitosan nanoparticles compared with ciprofloxacin antibiotic and composite from the two acid dyes with chitosan nanoparticles were studied carefully before being used as a safe material for textile finishing of hygiene materials. A549 cells were treated for 3 h and 24 h with chitosan nanoparticles (0–0.5 wt.%). The MMT method was used to investigate CSNP cytotoxicity in live A549 cells with reduced mitochondrial activity, as shown in (Figure 11).

Figure 11 shows that at lower concentrations of both CSNPs and ciprofloxacin, there are no toxicity effects of CSNPs on cells and low toxic effects of ciprofloxacin. At higher concentrations, CSNPs are still nontoxic whereas ciprofloxacin toxicity appears with 20% viable cells compared with about 98% viable cells for CSNPs. Therefore, it can be illustrated that there is no significant toxicity of CSNPs and they can be used as a safe material for textile finishing.

## 4. Conclusions

Chitosan nanoparticles (CSNPs) were used to finish acid-dyed cotton fabrics, which enhanced their functionality. CSNPs were produced using tripolyphosphate and chitosan ionotropic gelation. In this work, safe biopolymer chitosan nanoparticles in varying concentrations were applied to cotton fabric. Because chitosan nanoparticles could produce new cationic charges from the amino groups’ protonation on the cellulosic cotton textiles surfaces, they increased the dyeability of cotton fabrics with acid blue and acid red dyes colours. Following treatment with various CSNP concentrations, TEM imaging, and FT-IR spectroscopy of CSNPs, dyeability and antibacterial activity of AR/AB acid-coloured cotton fibres were examined. Following treatment with various CSNP concentrations, TEM imaging, and FT-IR spectroscopy of CSNPs, dyeability and antibacterial activity of AR/AB acid-coloured cotton fibres were examined. Cotton fabrics’ physic-chemical and mechanical properties have been enhanced by the high-power penetration of nanoparticles and physical crosslinking (presence of OH and NH_2_ groups in CSNPs moiety). The ideal concentration of chitosan nanoparticles for improving dyeability and antibacterial activity was 0.2 weight percent. Additionally, these coloured cotton fabrics contain antibacterial properties that protect against fungus, gram-positive, and gram-negative germs. For MTT finishing cotton fabrics, CSNPs are a secure, biocompatible, and environmentally friendly material.

## Figures and Tables

**Table 1:** Nitrogen content, mechanical properties, yellowness index, and whiteness index of untreated cotton fabrics, cotton fabrics treated with CSNPs, and cotton fabrics dyed with CSNPs.

Fabrics	Cotton Fabrics Treated with CSNPs				
	N %	E %	TS kg	YI	WI
Untreated cotton fabrics	0.0	11	48	1.59	54.37
Cotton fabrics treated with CSNPs	0.18	13	50	1.39	54.48
Acid Blue dyed cotton treated with CSNPs 317	0.22	14	52	1.25	54.97
Cotton treated with CSNPs and Acid Red 88 dyed	0.24	16	54	1.19	55.34

* N: nitrogen content; E: elongation at break; TS: tensile strength; WI: whiteness index; YI: yellowness index.

**Table 2:** Fastness properties of CSNPs treated cotton fabrics and dyed with two acid dyes at 0.2 wt.% nonionic detergent at 88 °C for 30 min.

CSNPs (wt.%)		Light Fastness	Washing Fastness			Perspiration Fastness						Rubbing Fastness	
						Alkaline			Acidic				
			Alt	SC	SW	Alt	SC	SW	Alt	SC	SW	Dry	Wet
0.1	AB	4–5	4–5	4	4–5	4–5	4–5	4–5	4–5	4–5	4–5	4–5	4–5
	AR	4–5	4–5	4	4–5	4–5	4–5	4–5	4–5	4–5	4–5	4–5	4–5
0.3	AB	5	4	4–5	4–5	4–5	4–5	4–5	4–5	4–5	4–5	4–5	4–5
	AR	5	4	4–5	4–5	4–5	4–5	4–5	4–5	4–5	4–5	4–5	4–5
0.5	AB	5–6	4	4–5	4–5	4–5	4–5	4–5	4–5	4–5	4–5	4–5	4–5
	AR	5–6	4	4–5	4–5	4–5	4–5	4–5	4–5	4–5	4–5	4–5	4–5

SC: staining on cotton; SW: staining on wool; Alt, colour change of dyed sample; AB: Acid Blue 317 dye; AR: Acid Red 88 dye.

## Data Availability

Data is contained within the article.
